# New data on the western Balkan leuciscids *Alburnoides* and *Alburnus* (Teleostei, Leuciscidae) from the Vjosa River, Albania

**DOI:** 10.3897/zookeys.870.36235

**Published:** 2019-08-07

**Authors:** Nina G. Bogutskaya, Harald Ahnelt

**Affiliations:** 1 First Zoological Department, Natural History Museum in Vienna, Burging 7, 1010 Vienna, Austria Natural History Museum in Vienna Vienna Austria; 2 Department of Theoretical Biology, University of Vienna, Althanstrasse 14, 1090 Vienna, Austria University of Vienna Vienna Austria

**Keywords:** Biodiversity, freshwater fish, range extension, southeastern Adriatic ecoregion

## Abstract

The first voucher-confirmed record of *Alburnus scoranza* and the first morphological description of *Alburnoides* for the Vjosa River system in Albania are reported with a brief discussion of the diagnostic morphological traits and taxonomic assignment of both species.

## Introduction

The Vjosa River (Aoos in Greece) belongs to the South Adriatic-Ionian zoogeographical division of Economidis and Bănărescu (1991) and is the southernmost river drainage of the Southeastern Adriatic ecoregion of Zogaris and Economou (2017). It originates in western Greece, flows through Albania, and drains into the southern Adriatic Sea. Some genera of the Greek (e.g., Economidis 1989, Durand et al. 1999, Economou et al. 2007, Geiger et al. 2014, Barbieri et al. 2017, Koutsikos et al. 2019) and the Albanian sections of the Vjosa/Aoos River (e.g., Šanda et al. 2008, Marková et al. 2010, Stierandová et al. 2016) were investigated mostly within wider phylogenetic studies, and summarising lists of fish species in the Albanian section have been also published (Ahnelt and Elvira 1991, Rakaj and Flloko 1995, Shumka et al. 2010, Graf et al. 2017, Meulenbroek et al. 2018, Shumka et al. 2018a,b, Weiss et al. 2018). However, any new data on morphology and distribution of the Vjosa fishes could help for further research, especially in taxonomically difficult groups or species complexes.

Herein, we report the first voucher-confirmed records of *Alburnoides* based on a historical sample and the Scoranza bleak *Alburnus scoranza* Bonaparte, 1845 present in the middle and lower part of the Vjosa River drainage in Albania; we also present first morphological data for the Albanian Vjosa populations of these two cyprinids. Their species-level identifications are discussed based on some morphological comparisons.

## Materials and methods

The Natural History Museum in Vienna (**NMW**) houses two small collections of freshwater fishes from the lower and middle course of the Vjosa River, from Selenica and Tepelena, respectively. These fishes were sampled in 1894 at the lower course of the Vjosa River near the town of Selenica (Selenicë) (Ahnelt and Elvira 1991) and in 1914 in the Bença River, a tributary of the Vjosa, near the village of Bença. No ecological data were available for either collection.

For methods and terminology of measurements and counts, and the comparative material, see Bogutskaya et al. (2010).

## Results

### Taxonomy

#### Alburnoides cf. devolli

Taxon classificationAnimaliaCypriniformesCyprinidae

(a member of the Alburnoides prespensis complex of Stierandová et al. (2016)

70b3338c-b898-5dd4-9a4c-3fad7dddd419

Fig. 1

##### New record.

NMW 55706, 40 specimens, SL 36.5–52.8 mm (mean 45.0 mm); Albania: Vjosa River drainage, Bença River at Bença village, ca. 6 km upstream of confluence with tVjosa (ca. 40°18'22"N, 20°1'29"E), collector(s) unknown, collected in July 1914, donated to NMW by F. Steindachner.

##### Identification.

Based on 40 specimens except for numbers of pharyngeal teeth and measurements as specified below. Dorsal-fin branched rays 8½; anal-fin branched rays 10½-12½ (mode 11½, mean 11.3½); scales in lateral series 45–51 (mean 48.1); total lateral-line scales 43–50 (mean 46.8); later-line scales to posterior margin of hypurals 43–49 (mean 45.8); ventral keel (n = 20) from completely to 1/3 scaled, commonly ¾ scaled; mouth with a fleshy snout protruding lower jaw; gill-rakers 7–9 (mode 8, mean 8); total vertebrae 39–41 (mode 40, mean 40.4) with abdominal vertebrae 20–21 (mode 20, mean 20.5), predorsal abdominal 13–15 (mode 14, mean 13.70) and caudal vertebrae 19–21 (mode 20, mean 19.9); most frequent vertebral formulae 20+20 and 21+20; pharyngeal teeth 2.5–4.2 (n = 5). Measurements for four specimens with SL over 50 mm see Table 1.

**Table 1.** Morphometric data of Alburnoides cf. devolli (member of *Alburnoides prespensis* complex of Stierandová et al. (2016) from river Bença (Vjosa drainage, Albania) deposited at NMW.

Alburnoides cf. devolli	NMW 55706: 1	55706: 2	55706: 3	55706: 4
Standard length (mm)	50.5	51.7	52.2	52.8
**Percent of standard length**				
Body depth at dorsal-fin origin	28.5	30.1	29.8	29.9
Body width at dorsal-fin origin	10.9	13.7	11.9	13.0
Predorsal length	54.6	55.7	57.2	56.4
Postdorsal length	37.9	36.3	35.8	37.5
Prepelvic length	49.5	50.7	50.9	48.7
Preanal length	66.9	68.7	69.3	65.2
Pectoral – pelvic-fin origin length	23.6	24.1	25.4	24.0
Pelvic – anal-fin origin length	19.0	18.3	15.4	17.3
Caudal peduncle length	23.9	21.8	22.9	24.3
Caudal peduncle depth	12.3	13.2	12.6	12.3
Caudal peduncle width	8.9	9.0	9.1	8.9
Dorsal-fin base length	11.6	12.3	11.4	11.7
Dorsal-fin depth	22.5	20.7	21.3	20.9
Anal-fin base length	13.6	14.5	14.6	13.3
Anal-fin depth	18.6	16.8	15.3	17.1
Pectoral fin length	21.0	20.4	20.2	20.5
Head length	26.4	26.5	25.6	24.9
Head depth at nape	18.8	19.2	18.2	18.8
Head width (maximum)	12.7	12.6	12.4	12.7
Snout length	7.6	7.4	7.5	7.5
Eye diameter (horizontal)	7.6	7.2	6.8	7.6
Postorbital distance	13.0	12.7	13.1	12.2
Interorbital width	8.8	8.3	8.8	8.8
Length of upper jaw	8.9	8.6	8.7	8.9
Length of lower jaw	11.3	11.1	10.0	11.3
**Percent of head length**				
Head depth at nape	71.1	72.4	76.0	73.0
Head width (maximum)	48.3	47.3	49.7	49.9
Snout length	28.7	28.1	29.4	30.0
Eye diameter (horizontal)	28.9	27.2	29.4	27.4
Postorbital distance	49.6	45.8	50.9	51.2
Interorbital width	33.5	31.3	33.6	35.2
Length of upper jaw	33.8	32.5	35.0	35.1
Length of lower jaw	42.7	41.9	43.2	40.3
Depth of operculum	40.3	39.9	38.2	40.5
**Percent of caudal peduncle length**				
Depth of caudal peduncle	51.3	60.7	53.2	52.0
**Percent of interorbital distance**				
Eye diameter (horizontal)	86.1	86.9	87.3	77.8

For a morphological comparison with close species (and presumptive species) see Table 2 and Fig. 2.

**Table 2.** Diagnostic characters for examined *Alburnoides* samples of North, Black, and Adriatic basins from the Rhine southwards to Vjosa in Albania. Modal values are in bold.

Identification sensu Stierandová et al. (2016)	Our identifications, including presumed species	Total lateral line scales	Anal fin branched rays	Total vertebrae	Abdominal vertebrae	Caudal vertebrae	Predorsal abdominal vertebrae	Most frequent vertebral formulae	Most frequent states of the ventral keel development as part of keel length covered by scales (scaled)
*A. bipunctatus* Lineage I	*A. bipunctatus*, Rhine (n = 22)	45–51; **48–50**; 48.4	13½–17½; **14½–15**½; [14.5]½	40–42; **41**; 41.4	20–21; **20**; 20.2	20–22; **21**; 21.2	13–15; **14**; 14.2	20+21	½ to ¾ scaled
*Alburnoides* sp. Lineage IV	A. cf. bipunctatus, Sava, upper Danube (n = 50)	45–54; **49–50**; 48.9	12½–15½; **13**½; [13.3]½	40–42; **41**; 41.1	20–21; **20**; 20.3	20–22; **21**; 20.8	13–15; **14**; 13.9	20+21, 21+21	2/3 scaled
*A. ohridanus* Lineage VII	*A. ohridanus*, Ohrid L. (n = 33)	42–46; **44**; 43.9	10½–13½; **11**½; [11.4]½	38–40; **39**; 39.0	19–21; **20**; 20.0	18–20; **19**; 18.9	12–14; **13**; 12.8	20+19	½ to ¾ scaled
–	A. cf. ohridanus, Skadar L. (n = 19)	42–47; **44**; 44.3	12½–15½; **12**½–**13**½; [12.8]½	39–41; **40**; 39.9	20–21; **20**; 20.3	19–20; **20**; 19.7	13–14; **14**; 13.8	20+20	¼ to 1/3 scaled
*A. prespensis* complex Lineage IX	*A. prespensis* Prespa L. (n = 3)	42–44; 43.0	10½–11½; **10**½; [10.3]½	**39**; 39.0	**20**; 20.0	**19**; 19.0	**13**; 13.0	20+19	¾ to completely scaled
*A. prespensis* complex *	*A. fangfangae* (n = 44)	6–53; **48–49**; 48.3	11½–14½; **12**½; [12.1]½	40–42; **40–41**; 40.6	20–21; **20**; 20.5	19–21; **20**; 20.1	13–14; **14**; 13.8	20+20, 21+20, 20+21	1/3 to 0 scaled (=completely scaleless)
*A. prespensis* complex *	*A. devolli* (n = 15)	44–48; **47**; 46.2	11½–13½; **12**½; [12.1]½	40–41; **40**; 40.3	20–21; **20**; 20.5	19–20; **20**; 19.8	12–13; **13**; 12.7	20+20	¾ to completely scaled
*A. prespensis* complex Lineage VIII	A. cf. devolli Vjosa (NMW 55760, n = 40)	43–50; **44–49**; 46.8	10½–12½; **11**½; [11.3]½	39–41; **40**; 40.4	20–21; **20**; 20.5	19–21; **20**; 19.9	13–15; **14**; 13.7	20+20; 21+20	½ to ¾ scaled

* Assignment to any lineage of Stierandová et al. (2016) cannot be determined without examination of voucher specimens.

**Figure 1.**
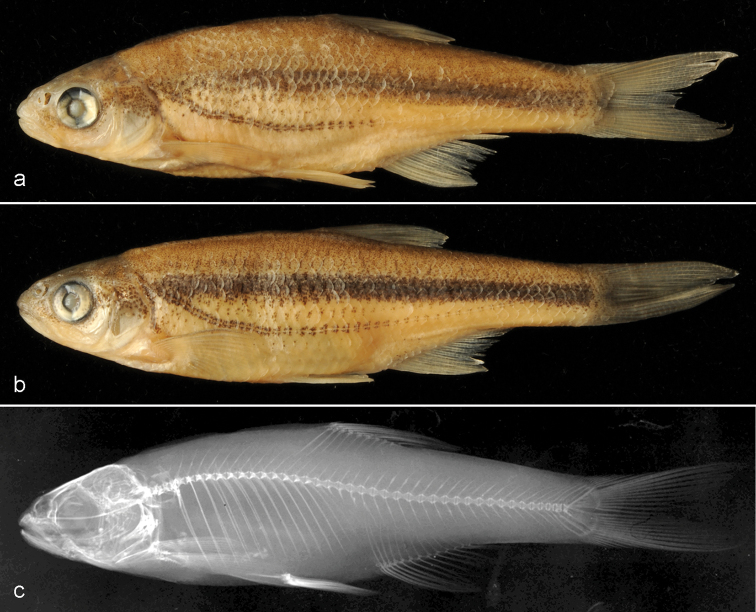
Alburnoides cf. devolli (member of *A. prespensis* complex of Stierandová et al. (2016), NMW 55706. External appearance of **a** male SL 50.5 mm and **b** female 47.9 mm, and **c** radiograph of same specimen as **a**.

**Figure 2.**
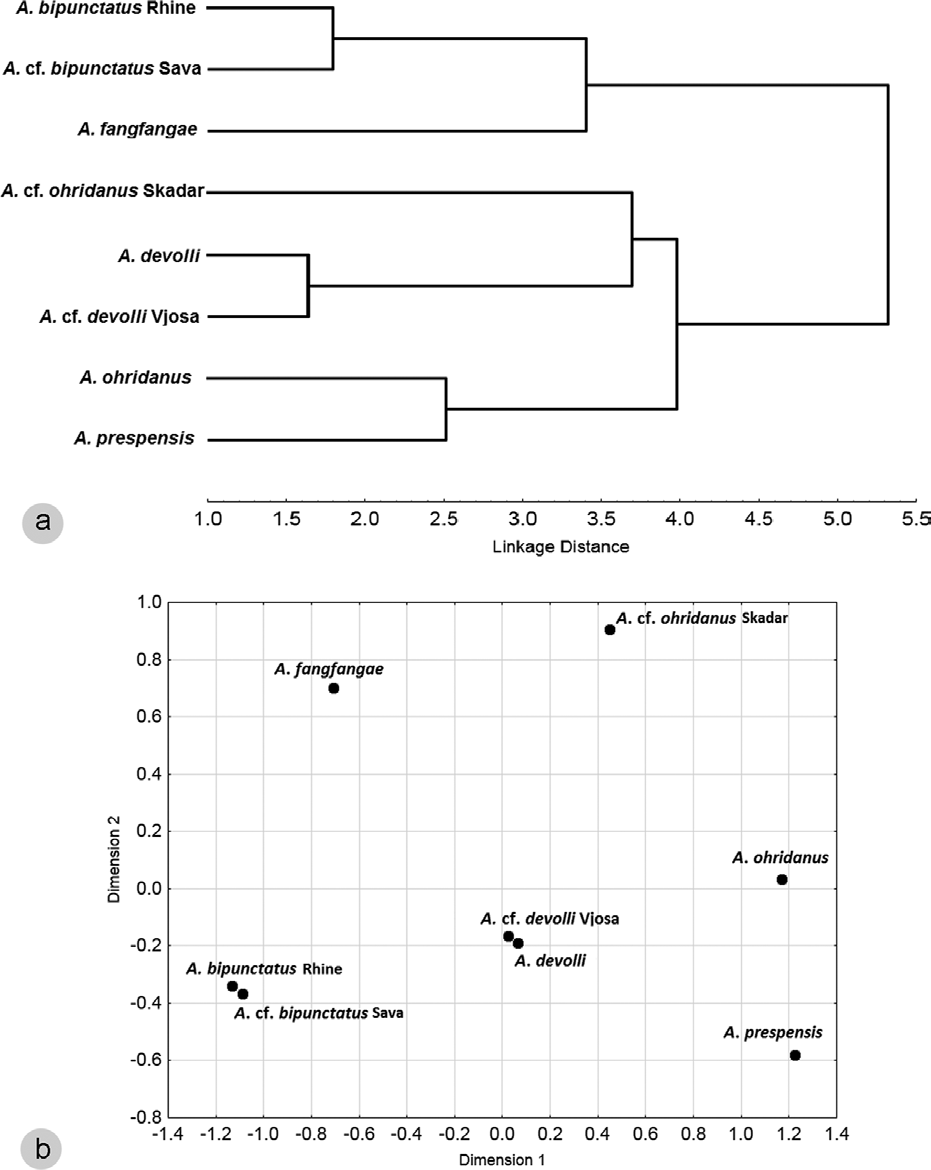
**a** Cluster analysis **b** multidimensional scaling results for *Alburnoides* samples of North, Black, and Adriatic basins from Rhine southwards to Vjosa in Albania based on data for counts and coded qualitative characters as in Table 3. Adriatic samples of *Alburnoides prespensis* complex named as presumed species.

Identification of the sample as Alburnoides cf. devolli is based on statistical analyses (Fig. 2). See Discussion for the taxonomy of *A. devolli* Bogutskaya, Zupančič et Naseka, 2012.

##### Colouration (preserved).

The body is pale fawn, the back darker than the ventral side. A distinct, black to dark grey stripe extends from the posterior margin of the orbit to the base of the caudal fin. The ventral side of the belly and of the tail is unpigmented. The lateral line is lined dorsally and ventrally by pigment along its entire length, most distinct in its anterior half to about below the origin of the dorsal fin. The fins are hyaline with tiny melanophores lining the dorsal and caudal fin rays, the anterior rays of the anal and the dorsal rays of the pectoral fins.

#### Alburnus scoranza

Taxon classificationAnimaliaCypriniformesCyprinidae

Bonaparte, 1845

AD3041ECC567595BB89248B578F6A4E4

Fig. 3

##### New record.

NMW 87654, 1 specimen, SL 70.8 mm; Albania: Vjosa River system: Selenica (40°32'N, 19°38'E); collected by Pola Expedition, Franz Steindachner, 4 July 1894. - NMW 87658-87659, 3 specimens, SL 90.8–125.6 mm; same data as NMW 87654.

##### Identification.

**Figure 3.**
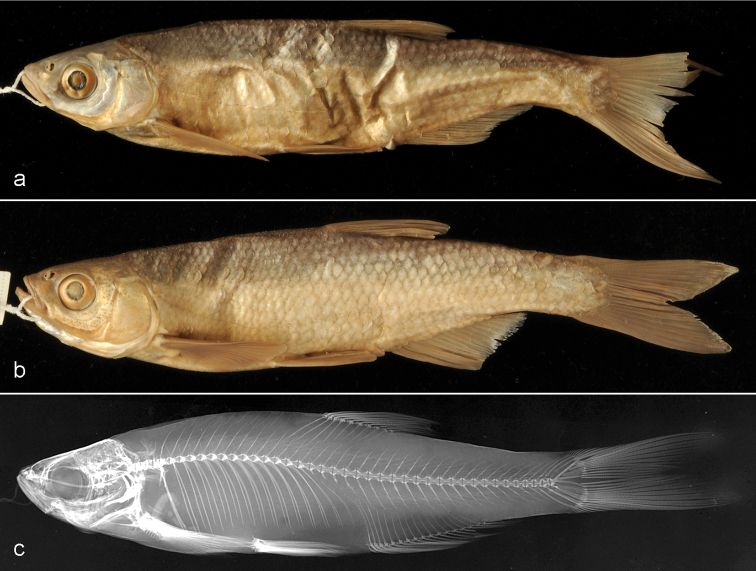
*Alburnus scoranza*, NMW 87659. **a** Female 106.9 mm SL **b** male 98.2 mm SL; NMW 87658 **c** radiograph, male 90.8 mm SL.

Measurements see Table 3. Dorsal-fin branched rays 8½; anal-fin branched rays 14½ (1 specimen) or 15½ (3 specimens); anal-fin origin behind base of 5^th^ (2), 6^th^ (1; Fig. 3c) or 7^th^ (1) branched dorsal-fin ray; mouth upturned, mouth cleft straight, tip of mouth about at level with upper margin of pupil; keel between pelvic fins and anus completely exposed, variably sharp; scales in lateral series 47–51 (mean 48.8); total lateral-line scales 45–51 (mean 48.3); later-line scales to posterior margin of hypurals 45–48 (mean 46.0); gill rakers 22–25 (23.5); total vertebrae 41 (22+19, 3 specimens) or 42 (22+20, 1 specimen) with predorsal abdominal vertebrae 15 (3 specimens) or 16 (1 specimen).

**Table 3.** Morphometric data of *Alburnus scoranza* from the Vjosa River at Selenize (Albania) and Montenegro (formerly considered syntypes, see text for discussion) deposited at NMW.

Alburnus scoranza	NMW 55700:1	55700:2	87654	87658	87659:1	87659:2
	female	female	female	male	female	male
Standard length (mm)	125.6	93.4	70.8	90.8	106.9	98.2
**Percent standard length**						
Body depth at dorsal-fin origin	23.3	18.2	19.9	24.0	22.9	23.8
Body width at dorsal-fin origin	10.8	8.7	8.5	7.8	8.2	8.5
Predorsal length (% SL)	57.8	57.6	58.4	54.9	56.0	54.0
Postdorsal length (% SL)	35.0	33.0	30.7	36.2	33.9	35.0
Prepelvic length (% SL)	47.7	48.9	45.7	43.6	47.1	46.1
Preanal length (% SL)	68.5	69.1	65.0	64.3	68.2	66.1
Pectoral – pelvic-fin origin length	24.5	23.6	23.0	20.9	23.8	21.1
Pelvic – anal-fin origin length	21.7	20.8	19.8	20.1	21.4	20.0
Caudal peduncle length	20.2	20.5	21.2	19.0	19.2	18.3
Caudal peduncle depth	9.6	8.5	10.3	11.0	10.6	10.0
Caudal peduncle width	7.0	5.6	5.7	5.1	5.5	5.7
Dorsal-fin base length	10.5	11.7	12.2	11.5	10.8	12.5
Dorsal-fin depth	16.4	18.8	20.5	20.9	19.6	21.1
Anal-fin base length	14.8	14.0	16.3	20.0	16.2	18.9
Anal-fin depth	12.7	15.3	13.4	14.9	12.4	15.5
Pectoral-fin length	18.1	20.2	20.3	21.9	21.5	22.6
Pelvic-fin length	13.8	15.9	14.4	17.6	15.8	18.3
Head length	23.8	24.7	25.5	23.7	24.4	25.1
Head depth at nape	15.1	15.5	16.9	15.6	15.7	17.7
Head width (maximum)	11.0	10.5	11.5	11.3	11.1	12.5
Snout length	5.3	6.3	6.6	6.3	6.9	7.0
Eye diameter (horizontal)	6.2	6.7	7.8	6.7	6.8	6.8
Postorbital distance	12.7	12.6	11.9	12.1	12.2	10.6
Interorbital width	6.7	6.6	7.3	7.4	7.5	7.4
Length of upper jaw	6.0	6.4	7.7	7.2	7.5	6.9
Length of lower jaw	8.4	9.3	9.4	9.6	9.2	9.6
**Percent head length**						
Head depth at nape	63.3	62.7	66.1	65.9	64.2	70.3
Head width (maximum)	46.2	42.3	44.9	47.6	45.3	49.7
Snout length	23.3	25.5	25.9	26.6	28.2	27.9
Eye diameter (horizontal)	25.9	27.0	30.7	28.3	28.0	27.2
Postorbital distance	50.5	50.8	46.5	51.2	46.5	48.5
Interorbital width	28.3	26.5	28.4	31.0	30.7	29.6
Length of upper jaw	25.1	25.8	30.0	30.2	30.8	27.4
Length of lower jaw	35.2	37.5	37.6	39.5	37.6	38.3
Depth of operculum	36.9	37.2	38.2	37.3	36.5	37.0
**Percent caudal peduncle length**						
Depth of caudal peduncle	47.3	41.3	44.0	48.6	52.2	54.6
**Percent interorbital distance**						
Eye diameter (horizontal)	91.5	101.6	107.8	91.3	91.1	91.9

##### Sexual dimorphism.

Though only few specimens were examined, we may report on pronounced sexual dimorphism: the pectoral fin is longer while the pectoral-pelvic distance is shorter in males and the pectoral fin reaches behind the origin of the pelvic fin (Fig. 3a, b). Also, the pectoral fin is markedly rounded with a slightly convex outer margin and the first two branched rays of about similar length forming the apex of the fin in males vs. a clearly pointed fin with a slightly concave outer margin and the first branched ray which is clearly longer than the second one forming the apex of the fin in females. The male NMW 87659:2 has prominent nuptial tuberculation: tubercles densely cover the entire dorsal and lateral head surface down to the lower margin of the interoperculum and the branchiostegal rays, anterior back along the scale margins and on the upper surface of the pectoral fin along all its rays.

##### Colouration (preserved).

The dorsal part of the head and the body is dark grey. The flank and the abdomen are silvery. The fins are hyaline.

## Discussion

As the knowledge of the biodiversity in general and of the fish fauna in particular within the Vjosa River system in Albania is very scarce (Rakaj and Flloko 1995, Shumka et al. 2018b), descriptions of fishes from this river system are commonly based on the study of Rakaj (1995). However, the Rakaj’s data were mostly based on material from Prespa, Ohrid, and Skadar lakes and their tributaries. This author did not give data for specimens of the Vjosa just indicating the occurrence of a species in this river.

Recent faunal lists mentioned the occurrence of an *Alburnoides* Jeitteles in the Vjosa River drainage under the names either *A. bipunctatus* (Bloch, 1782) (Meulenbroek et al. 2018, Shumka 2018b) or *A. prespensis* (Karaman, 1928) (Shumka et al. 2018a). Morphological (Coad and Bogutskaya 2009, Bogutskaya et al. 2010) and molecular (Geiger et al. 2014, Stierandová et al. 2016) data ruled out the occurrence of *A. bipunctatus* in the Vjosa/Aoos River (Barbieri et al. 2015, 2017). Mentioning *A. prespensis* in the Vjosa River in Albania, Shumka et al. (2018a) just refer to previous authors, e.g., Rakaj (1995), Kottelat and Freyhof (2007), Bogutskaya et al. (2010) or Geiger et al. (2014). Whereas Rakaj (1995) and Kottelat and Freyhof (2007) do not give records of *A. prespensis* outside of the Prespa lakes and their tributaries, the other authors just refer to records in the Greece part (Aoos) of the Vjosa River drainage.

Limited mitochondrial (cytb) data of Perea et al. (2010) – only one specimen from Prespa Lake and one from the Aoos were studied – showed a very close position of the two specimens. Results of Geiger et al. (2014) clearly demonstrated that the CO1 marker did not provide resolution in many groups of conventional species in the subfamily Leuciscinae (Geiger et al. 2014: table S1-C). This table lists at least 17 complexes of “closely related” leuciscine species, which include clearly morphologically distinct species, e.g., *Delminichthys ghetaldii* (Steindachner, 1882), *D. jadovensis* (Zupančič et Bogutskaya, 2002) and *D. krbavensis* (Zupančič & Bogutskaya, 2002) or *Squalius tenellus* Heckel, 1843 and *S. microlepis* Heckel, 1843. *Alburnoides prespensis*, *A. devolli* and *A. fangfangae* Bogutskaya, Zupančič & Naseka, 2012 were not distinguished by COI barcode either: *A. devolli* and *A. fangfangae* from the Seman drainage were closely related to *A. prespensis* from Prespa Lake and the position of this cluster was a nearest sister to *A. ohridanus* (Karaman, 1928) (Skadar material was not examined) (Geiger et al. 2014). A study using a set of both nuclear (ß-actin, RAG1 and S7) and mitochondrial (cytb) markers (Stierandová et al. 2016) supported the divergence of the “*prespensis+devolli+fangfangae*” cluster from *A. ohridanus* (lineage VII) but subdivided most part of the Albanian samples into three lineages (VII, IX and X) naming them “*A. prespensis* species complex”. This term has been in use since then (e.g., Juladeh Roudbar et al. 2016, Barbieri et al. 2017).

In the publication by Stierandová et al. (2016), the *A. ohridanus* lineage included the Ohrid Lake sample and some individuals from the Mat, Ishëm and Erzen Rivers - Adriatic drainages south of the Ohrid-Drin-Skadar basin. The range of the *A. prespensis* species complex embraces Prespa Lake and five Adriatic drainages in the south of Erzen – Shkumbin, Seman, Vjosa/Aoos, Dukati, and Borshi. The internal structure of the *A. prespensis* complex based on combined data (cytb, ß -actin and S7) (Stierandová et al. 2016: fig. 1) cannot be interpreted from either taxonomic or zoogeographic aspects. As any morphological diagnostic characters of the voucher samples used for the genetic research have not been published, a clear taxonomic assignment of the Aoos (Greek) *Alburnoides* as well as of the two Albanian species from the Seman River drainage (geographically closest to the Vjosa/Aoos) is still uncertain.

Our morphological data (Table 2, Fig. 2) is not congruent with the molecular data: *A. ohridanus* is located inside the *A. prespensis* complex and the whole set of samples that belong to the latter demonstrates a high degree of morphological divergence within the group in general and between some presumptive species, in particular. However, as it can be seen from Table 3 summarising our data for samples out of the *A. prespensis* complex, they are all different in key diagnostic characters indicating a considerably high morphological diversity of the complex. Our sample is similar to samples from the Aoos in Greece (Barbieri et al. 2017) in having a poorly developed ventral keel which is commonly almost or completely scaled, and 43–49, mean 45.8, lateral-line scales to posterior margin of hypurals (45–50, mean 46.3 in Barbieri et al. (2017). However, the number of branched anal-fin rays is lower, commonly 11½, mean 11.2 [½] (vs. commonly 12½, mean 11.8[½] in Barbieri et al. (2017).

The entire *A. prespensis* complex needs a thorough revision, especially with regard to the southern river drainages of Albania (Bogutskaya et al. 2010, Stierandová et al. 2016, Barbieri et al. 2017). The data presented in this study are a first step towards resolving the phylogenetic and taxonomic position of the Vjosa/Aoos A. cf. devolli populations.

The Vjosa population of *A. scoranza* in Albania was documented as *Alburnus alburnus* (Linnaeus, 1758) by Ahnelt and Elvira (1994). The Aoos population of *A. scoranza* in Greece was also first reported as *A. alburnus* by Economou et al. (2007a) and then as A. cf. scoranza (Economou et al. 2007b). *Alburnus scoranza* is supposedly distributed in the western Balkans from the Drin drainage, including Skadar and Ohrid lakes, south to the Aoos in Greece where it is restricted to a short section of the middle section of the river within the Konitsa plateau downstream to the Albanian border (Barbieri et al. 2015). Based on COI barcodes, Geiger et al. (2014) showed that *A. scoranza* (material from Ohrid and Skadar lakes and the Aoos in Greece) is not included in the Adriatic *Alburnus* clade but is the closest neighbour to a wide group of *Alburnus* species spread from Portugal and France to Central Anatolia. Mangit and Yeril (2018) included some GenBank-available CO1 sequences of *A. arborella* (De Filippi, 1844), *A. albidus* (Costa, 1838), *A. belvica* Karaman, 1924, and *A. scoranza* in their analysis of mostly Turkish species and found the same pattern with *A. scoranza* as a closest sister clade to the three former species plus two from western Turkey.

While *A. arborella* occurs in northern Adriatic basin, the distribution of *A. belvica* is restricted to Prespa Lake with its tributaries (Kottelat and Freyhof 2007). *Alburnus neretvae* Buj, Šanda et Perea, 2010 is an endemic species of the Neretva River drainage (Buj et al. 2010) and the distribution area of *A. scoranza* comprises the basins of lakes Prespa, Ohrid, and Skadar (Kottelat and Freyhof 2007). Economou et al. (2007b) and later Geiger et al. (2014) listed this species for the Greek part of the Vjosa/Aoos River drainage. Shumka et al. (2018a) mentioned *A. scoranza* in a checklist for the Albanian section of the Vjosa but no exact locality data were given. Therefore, the specimens presented in this study from Selenica are the first voucher-confirmed record of *A. scoranza* for the Vjosa River system in Albania.

We could only morphologically compare our sample with the limited published data of *A. scoranza* (Buj et al. 2010; material from the Zeta, Skadar basin; Black Drin, Ohrid basin; and the Mat River, which is the geographically closest drainage south of the Drin). The Vojsa specimens have more numerous anal-fin branched rays, 14½–15½ (vs. 13½–14½); similar number of total lateral-line scales, 45–51 (vs. 46–53); and, the most striking difference, gill rakers 22–25 (vs. 15–20). Interestingly, the Vojsa sample does not differ by the diagnostic counts from two specimens that had been considered syntypes of *A. scoranza* Heckel & Kner, 1857 from Skadar Lake (NMW 55700: 1 and 2) until it was supposed that the species’ name was made available earlier by Bonaparte (1845) (see below). These two specimens have a sharp scaleless keel; total lateral line scales 48, 47; anal-fin branched rays 14½; gill rakers 21, 24; vertebrae 22+19 and 22+20 with predorsal abdominal vertebrae 15 and 16; for measurements see Table 2.

So far, clarification of the taxonomic status of *A. scoranza* from the Vjosa still needs additional morphological and genetic data, especially from the southern river basins in Albania.

**Nomenclatural note on *Alburnus scoranza*.** The most recent publications (e.g., Buj et al. 2010; Barbieri et al. 2015) follow Kottelat and Freyhof (2007: 598) in attributing the authorship to Bonaparte (1845). Bonaparte (1845: 12) lists *A. scoranza* (species No 122) with a reference to Heckel (no date) and the only morphological trait given in the description is the number of pharyngeal teeth (2.5–5.2). According to Art. 12.1 of the International Code of Zoological Nomenclature (the Code; International Commission on Zoological Nomenclature 1999) this indeed constitutes an available name as it is “accompanied by a description … of the taxon that it denotes”. Heckel’s publication is most probably dated 1843 where *Alburnus scoranza* is listed (on page 1036) as a name only (authorship attributed to Heckel) with the locality as Montenegro, among other species names under the description of the genus *Alburnus*. The pharyngeal teeth 2.5–5.2 are given as a characteristic feature of the genus. This may indicate that Bonaparte only referred to these data from Heckel (1843) and did not examine any *A. scoranza* specimens himself, so, Heckel’s specimens may represent the type series of the species by bibliographic reference according to Art. 72.4.1 of the Code (International Commission on Zoological Nomenclature 1999).

Heckel had apparently examined specimens of *A. scoranza* as two specimens (as *Aspius scoranza* Heckel, acquisition number 1843.II.18b) were registered at the NMW from “Cettinje in Montenegro” collected by himself in his 1840 travels. Unfortunately, these specimens have not been found in NMW during recent searches. Cetinje is a city (the historic old capital of Montenegro) located in the Cetinje karst field 12 km from Skadar Lake and even closer to the Rijeka Cernojevića River, an inflow of Skadar Lake. Heckel received two more specimens (acquisition number 1856.VII.26) described by Heckel and Kner in 1857 (page 139 footnote) much later than 1843 from Belotti (sample NMW 55700). In case a designation of neotype to fix the species name *A. scoranza* is needed in the future, the information presented above on the Heckel’s specimens from Cetinje should be taken into consideration to meet the conditions of Art. 75.3 of the Code (International Commission on Zoological Nomenclature 1999).

Recent studies provide evidence that species of *Alburnoides* and *Alburnus* Rafinesque were introduced into other river systems (e.g., Simić et al. 2012, Stierandová et al. 2016, Pofuk et al. 2017, Vukić et al. 2019). As intentional and unintentional introductions of non-native fish species are common in Adriatic lake and river systems (e.g., Shumka et al. 2008, Simić et al. 2012, Piria et al. 2017, Pofuk et al. 2017, Vukić et al. 2019), historic museum collections provide important information of natural fish distributions (e.g., Palandačić et al. 2017). If several phylogenetic lineages occur in one and the same river drainage, historic museum collections may be of a crucial importance to determine native populations and apply the nomenclaturally correct name.

## Supplementary Material

XML Treatment for Alburnoides cf. devolli

XML Treatment for Alburnus scoranza
